# Supplementary material for: A network dynamical simulation model for the study of antibiotic resistance in microbial communities
Source: Gut Microbes. 2026 Sep 19;18(1):2734703. doi: 10.1080/19490976.2026.2734703 (PMC13596919; doi:10.1080/19490976.2026.2734703)
Supplement: Supplementary file 1 [file KGMI_A_2734703_SM9053.zip › supplementary_file_1/Figures/real_delta_gc_frac.pdf]

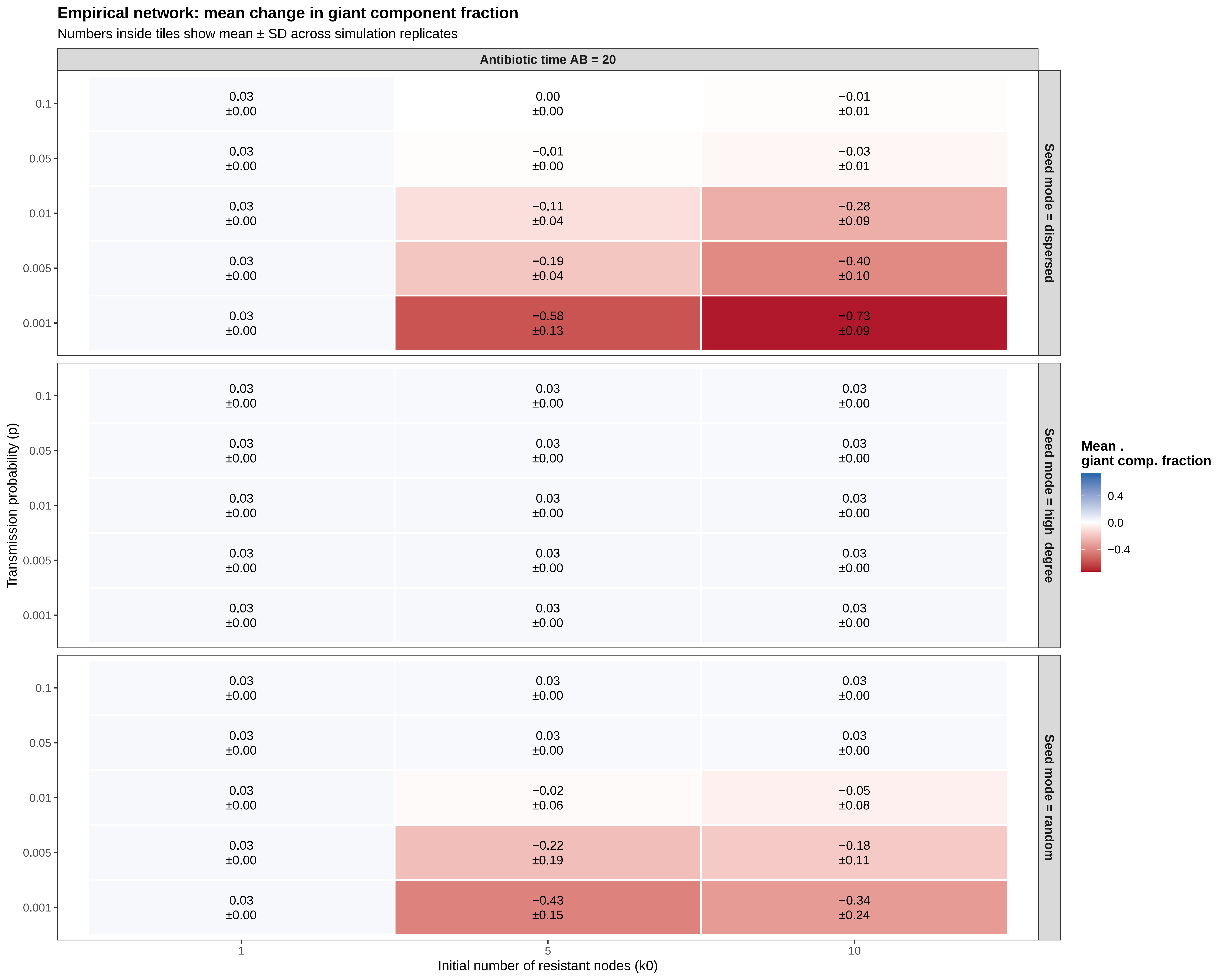

Empirical network: mean change in giant component fraction
Numbers inside tiles show mean ± SD across simulation replicates
Antibiotic time AB = 20
Seed mode = dispersed
Seed mode = high_degree
Seed mode = random
Transmission probability (p)
Initial number of resistant nodes (k0)
0.1
0.05
0.01
0.005
0.001
1
5
10
0.03 ±0.00
0.00 ±0.00
−0.01 ±0.01
−0.01 ±0.00
−0.03 ±0.01
−0.11 ±0.04
−0.28 ±0.09
−0.19 ±0.04
−0.40 ±0.10
−0.58 ±0.13
−0.73 ±0.09
−0.02 ±0.06
−0.05 ±0.08
−0.22 ±0.19
−0.18 ±0.11
−0.43 ±0.15
−0.34 ±0.24
Mean .
giant comp. fraction
0.4
0.0
−0.4
